# Retrospective study of the bone morphology in the posterior mandibular region. Evaluation of the prevalence and the degree of lingual concavity and their possible complications

**DOI:** 10.4317/medoral.21256

**Published:** 2016-10-01

**Authors:** Judit Herranz-Aparicio, José Marques, Nieves Almendros-Marqués, Cosme Gay-Escoda

**Affiliations:** 1 DDS. Master degree program of Oral Surgery and Implantology, School of Dentistry, University of Barcelona; 2 DDS, MS. Master degree program of Oral Surgery and Implantology, School of Dentistry, University of Barcelona; 3 DDS, MS. Associate Professor of Oral Surgery. Professor of the Master of Oral Surgery and Implantology. University of Barcelona Dental School. Investigator of the IDIBELL Institute; 4 MD, DDS, MS. PhD, EBOS. Chairman and Professor of Oral and Maxillofacial Surgery. Director of the Master of Oral Surgery and Implantology. Barcelona University, School of Dentistry. Coordinating/ Investigator of the IDIBELL Institute. Head of the Department of Oral and Maxillofacial Surgery and Coordinator of the Orofacial Pain Unit, Teknon Medical Center.Barcelona, Spain

## Abstract

**Background:**

In order to choose the appropriate implant size and to prevent complications, an oral surgeon must know the size and shape of the mandible. In the posterior mandibular region, a lingual undercut is often found and could represent a difficulty hard to manage if a lingual or buccal perforation occur. A large series of computed tomography (CT) images of the mandibular first molar was evaluated and the bone morphology, the prevalence and the degree of the lingual concavity in the first molar region were studied.

**Material and Methods:**

One hundred and fifty-one computed tomography (CT) examinations of patients were retrospectively evaluated to determine anatomical variations in bone morphology in the submandibular fossa region.

**Results:**

A total of 151 subjects were included, consisting of 64 males (M) (42.4%) and 87 females (F) (57.6%). The under-cut type ridge was present in 64.2% of the cases. The concavity angle was 66.6 ± 8.9° (M) and 71.6 ± 8.4° (F) and the linear concavity depth 4.5 ± 2.3 mm (M) and 3.1 ± 1.7 mm (F) (*p*>0.05).

**Conclusions:**

Mandibles with any lingual concavity present a potential increased risk of lingual cortical perforation during implant placement surgery. CT imaging allows characterizing the anatomy of the submandibular fossa and provides other important information for the preoperative assessment of the posterior mandible for dental implants placement.

**Key words:**Anatomy, computed tomography, dental implants, intraoperative complications, mandible, panoramic radiography, radiographic examination.

## Introduction

In oral implantology, the most serious and frequent complications described in the literature occur during surgery. They may result from inadequate planning, overworking of the implant bed, contamination of the implant by incorrect manipulation or mishandling, by poor implant orientation, or by the surgical procedure itself, which is not without risk (1).

Precise evaluation of bone size and morphology, with clinical examination and palpation of the bone ridge at the implant site, is essential for preoperative planning of mandibular implant placement (2,3). Furthermore, the size of the selected implant depends on the height and width of available bone and on the location of mandibular canal (3). In the posterior region the mandibular canal and the concavity of the submandibular fossa may limit available bone, leading to potential complications (2).

The intraoperative complications related with implant mandibular surgery are hemorrhages, neurosensory alterations, damage of the teeth adjacent to the implant, and mandibular fractures (1).

The submandibular fossa is a depression on the medial surface of the mandible inferior to the mylohyoid line. It contains the submandibular gland and the sublingual gland is found in the sublingual fossa. This fossa is a shallow depression on the medial surface of the mandible on both sides of the mental spine, superior to the mylohyoid line. The submandibular and sublingual fossae must be palpated prior to osteotomy development (4). Mandibular posterior lingual concavity (LC) is a common clinical finding, and the risk of perforation is high especially when the fossa is very deep (5,6). During the implant treatment or others surgical procedures, including extractions, osteotomies, periodontal surgery involving osteoplasty, biopsies of the floor of the mouth and the bone augmentation techniques, such as lateral and onlay graft or osteodistracction of the mandible, also have a potential for vascular injury and subsequent bleeding (7-9). Inadvertent instrumentation through the lingual cortical plate (LCP) can lead to arterial trauma, which may result in the development of a sublingual o submandibular hematoma, excessive bleeding, or infection (2,5).

The aim of this retrospective study was to evaluate a large series of CT images of mandibular first molar and to study the bone morphology, the prevalence and the degree of the LC in the first molar region.

## Material and Methods

A cross-section study of 151 CTs between 2010 and 2011 in the Oral Surgery and Implantology Department of the School of Dentistry of the University of Barcelona (Barcelona, Spain), was carried out. The CTs were taken for diagnostic assessment purposes for oral surgery and implant-supported rehabilitation.

Selection criterion was: visualization of the posterior mandibular region, a minimum of 3.5mm of horizontal bone width. The CTs were excluded if the images presented artifacts, if bone pathology was present in the region of the first mandibular molar, cases with grafted alveolar ridge or implants placed in this position.

In order to also consider immediate implant cases, both dentulous and edentulous first mandibular molar spaces were included.

All the CTs were acquired on a GE Lightspeed 5x scanner (GE Healthcare, Buckinghamshire, United Kingdom). The operation parameters were 150 mA, 120 kV and exposure time 10 seconds.

The present study was independently reviewed and approved by the Clinical Research Ethics Committee (CEIC) of the Bellvitge University Hospital (Barcelona, Spain). Written consent of each subject was signed prior to the evaluation.

The evaluation of the mandibular lingual concavity was performed by a simple researcher. All CTs were viewed on a radiologic view box, using a caliper and a protactor, and were assessed under standardized conditions.

Mesiodistally, if the second premolar and molar were present, the cross-sectional view of the middle of the edentulous ridge was selected, otherwise, it was selected the one 5 mm distal to the second mandibular premolar.

**Figure 1:**
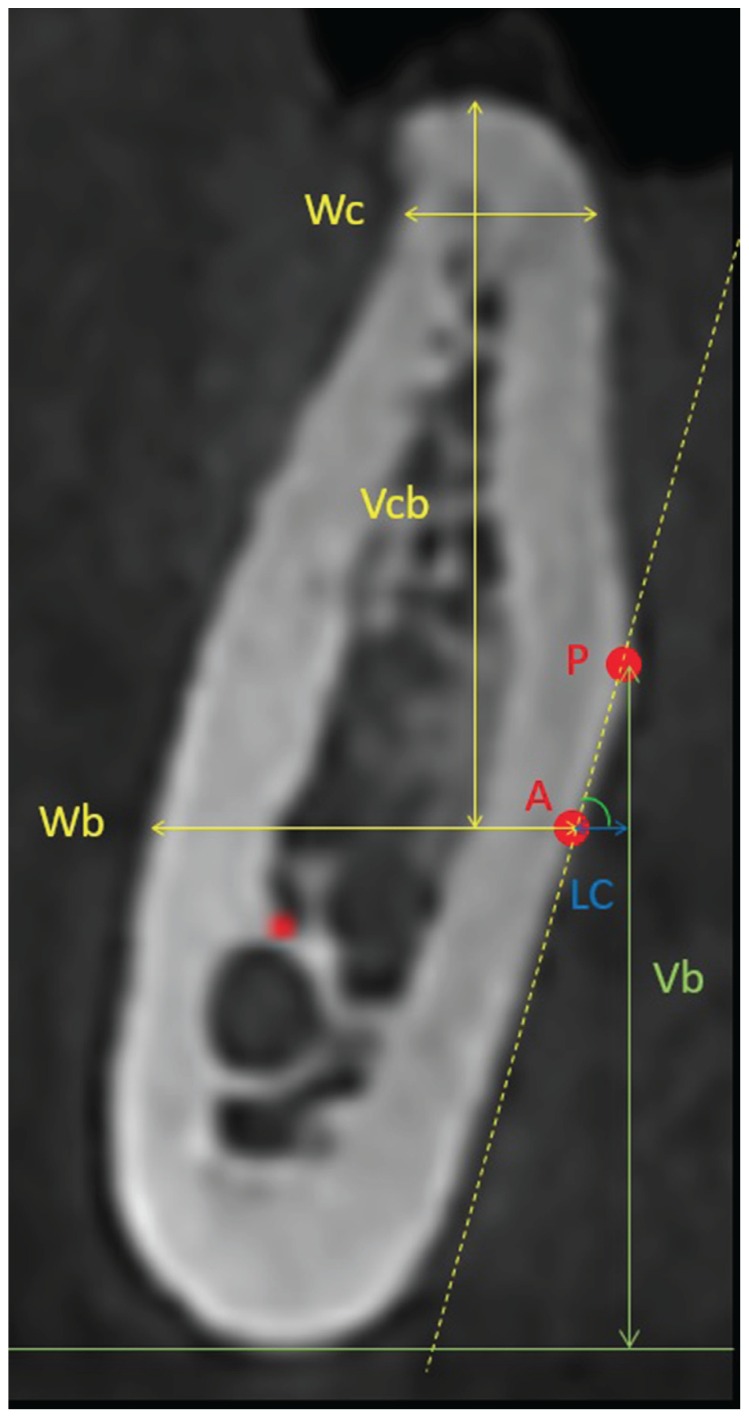
Demonstration of cross-sectional view showing the relevant measurements.

The mandibular cross-sectional morphology 2mm coronal to the inferior alveolar nerve was categorized into one of the following three groups: a ridge with a narrow base that expands bucco-lingually to wider crest with a prominent point on the lingual plate (point P), giving rise to a lingual undercut, was classified as an undercut ridge (U), when no obvious lingual undercut was seen, the ridge was categorized into either the convergent ridge type (C) or the parallel ridge type (P). The type-C ridge was one where the base of the ridge was wider than its crest. On the other hand, the type-P ridge generally had a more or less parallel ridge form. The concavity angle, in degrees, was determined by the angulation between line a and line b (the connection of point A and point P). The linear concavity depth (D) was also measured as the horizontal distance between point A and point P. The bigger the concavity, the smaller the angle and the bigger the depth were. (Fig. 1)

On the selected cross-sectional image, the line a was defined as a horizontal line 2mm coronal to the superior border of the inferior alveolar nerve (IAN). Point A was determined by the intersection between line A and the lingual plate. For morphologic characterization of this area, the bucco-lingual width 2mm apical to the alveolar crest (Wc) and at the level of line A (Wb) was measured. The vertical distance from alveolar crest to line A (Vcb) and the distance from point P to the inferior mandibular border (Vb) was also measured. (Fig. 1)

The data obtained were analyzed using SPSS 19.0 statistical package (SPSS Inc., Chicago, IL, USA). When the distribution was compatible with normality, the mean, standard deviation (SD) and an independent samples t-test was used. A non-parametric independent samples Mann-Whitney test was also calculated. A Pearson correlation coefficient was used for continuous variables. The level of significance was set at *P*<0.05 and a 95% confidence interval was calculated.

## Results

A total of 151 subjects were included, consisting of 64 males (M) (42.4%) and 87 females (F) (57.6%) with a mean age of 42.3±15.4 (M) and 39.33±12.8 (F), respectively. The mandibular size is summarized in  Table 1. The bucco lingual width 2 mm apical to the alveolar crest (Wc) was 10.6± 2.1 mm (M) and 9.7±1.6 mm (F) and the difference between genders was not significant (*p*>0.05). The mandibular width 2 mm coronal to IAN (Wb) was 11.8±2.7 mm (M) and 11.49±2.1mm (F) (*p*<0.05). The vertical height from alveolar crest to 2 mm coronal to IAN (Vcb) was 14.6±2.8 mm (M) and 13.5±2.4 mm (F) (*p*>0.05). The U type was present in 64.2% (n=97), the second was the P group presented in 22.5% (n=34), and the type C was only present in 13.2% (n=20).

**Table 1:**
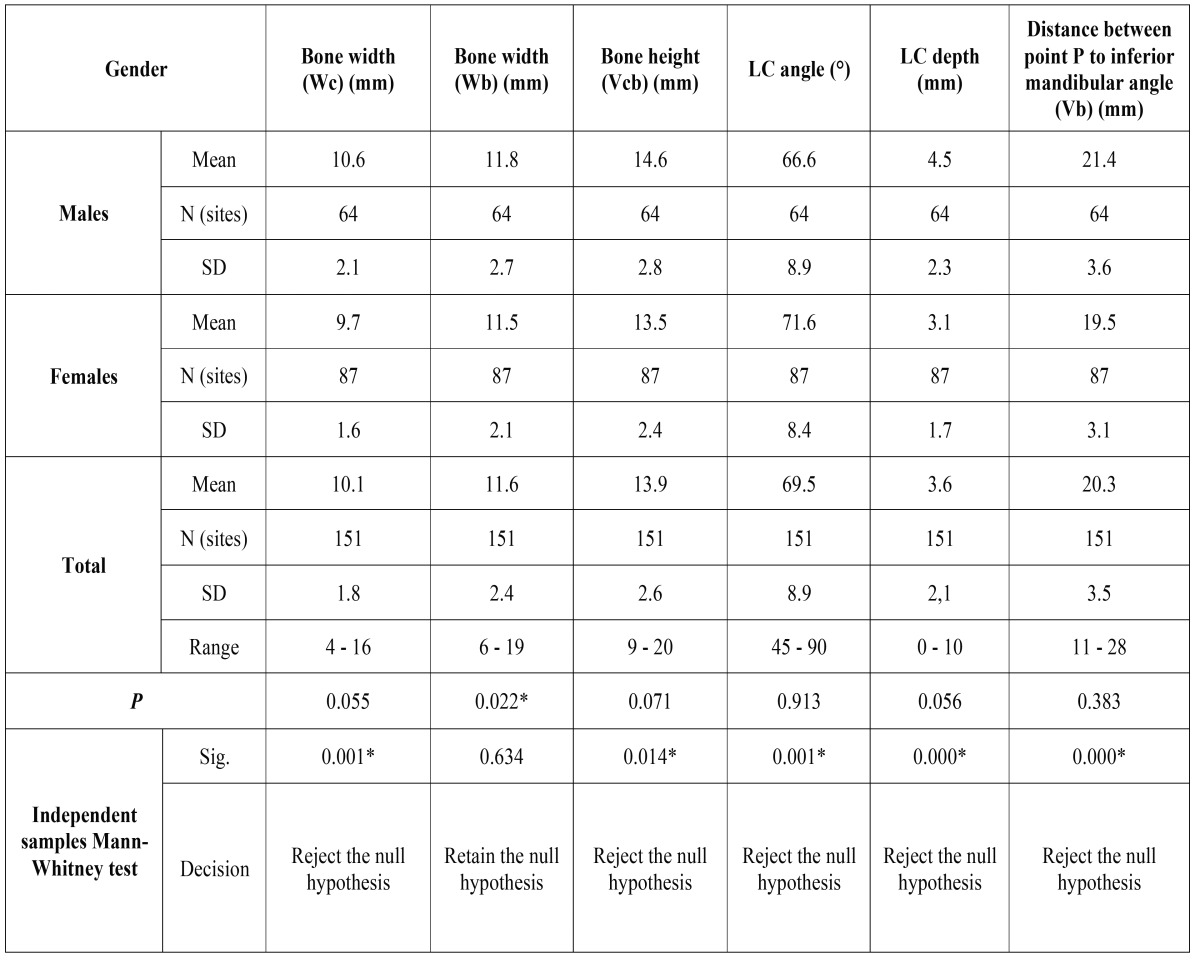
Measurements of mandibular dimension and lingual concavity.

The concavity angle was 66.6±8.9° (M) and 71.6±8.4° (F) and the linear concavity depth (LC) 4.5±2.3 mm and 3.1±1.7 mm (*p*>0.05). The vertical distance from alveolar crest to line A (Vcb) was 14.6±2.8mm (M) and 13.5±2.4mm (F) (*p*<0.05) and the distance from point P to the inferior mandibular border (Vb) was 21.4±3.6mm (M) and 19.5±3.1mm (F). ( Table 1)

A negative correlation was found between age and the bucco lingual width 2 mm apical to the alveolar crest (Wc) and also between age and linear concavity depth (LC) (*p*<0.05). ( Table 2)

**Table 2:**
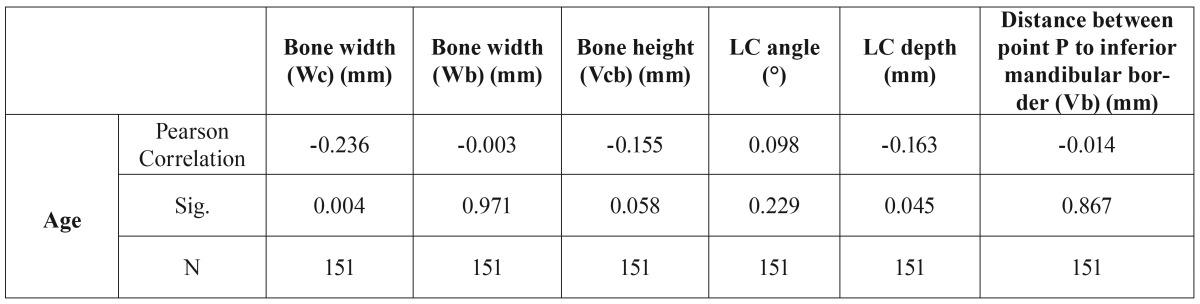
Correlation between age and the measurements of mandibular dimension and lingual concavity.

## Discussion

An oral surgeon must know the size and shape of the mandible to select the appropriate implant size and to prevent complications. To prevent lingual or buccal perforation during drilling, the implant should be installed according to the shape of the mandible (3). Authors like Parnia *et al.* (6), Chan *et al.* (5) and Watanabe *et al.* (3) studied the size and shape of the mandible using a computed tomography (CT) or a cone-beam computed tomography (CBCT). Chan *et al.* (5) and Parnia *et al.* (6) reported higher prevalence of a type U mandible (66%) in comparison to the type P or C, while Watanabe *et al.* (3) reported that the undercut type was only present in 39% of the cases. Other authors, such as Huang *et al.* (10) and Nickenig *et al.* (11) observed similar prevalence of undercut type mandible in the first molar area (56.2% and 56% respectively). These authors also concluded that the mandibles with a lingual concavity, the type U mandibles, present a potential increased risk of LCP. According to our results the type U was the most commonly seen with 64.2% of the cases falling into this category. The differences observed in these studies might be attributed to the different classification used, or the ethnicity of the studied population (Japanese in Watanabe’s study, compared to mostly Caucasians and African-Americans in the Chan’s study, Taiwanese in Huang’s Study or Caucasians in Nickenig’s and in our study). Huang *et al.* and Nickening *et al.* also found an increase of the undercut type prevalence when the second molar area was evaluated. (10,11)

The difference between genders was not significant concerning Wc, Vcb, Lc angle and LC depth (*p*>0.05). Nevertheless, Wb was significantly wider in males (*p*<0.05). Chan *et al.* (5) found no differences between genders regarding Wb values, in contrast, a wider Wc in males was reported (*p*<0.05). Differences in the assessment method of the cross-sectional morphology of the mandible make very difficult to compare the measurements obtained in the different studies.

Froum *et al.* (2) and Chan *et al.* (5) studied the risk of LCP in implant treatment in the posterior mandibular region. Both used a cone beam computerized tomography (CBCT) and a computerized tomography (CT), respectively, and software that allowed the virtual installation of 4-5 mm diameter implants with a variable length (depending on the height of the alveolar crest). The mandibular first molar was present in the first study and missing in the second one. These authors concluded that the risk of LCP during implants surgery is higher in those cases with a marked LC. Froum *et al.* (2) recommended pre-extraction CT to assess the risk of the inferior alveolar nerve (IAN) injury and lingual plate perforation for immediate implant placement in the posterior mandible, in order to be able to assess other options like the delayed protocol.

Most reports of hemorrhage in the floor of the mouth and sublingual hemorrhage have been caused by an iatrogenic procedure (6). The hemorrhages in the mandible occur most frequently in the interforaminal region, especially in the canine region (9), and recently various cases have reported LCPs and arterial damage of this region with consequently respiratory obstruction (8,12,13). Sublingual hematoma formation after implant placement is an uncommon complication, but it is potentially life-threatening (6,8).Hemorrhage caused by damaging the sublingual arteries during implant surgery depends on the anatomy of the bone at the implant site, the diameter of the vessel, and/or the distance of the vessel to the bone. In atrophic edentulous mandibles this distance is even shorter and perforations become deeper in the floor of the mouth (6,12).

In the posterior region the main complications are related to the lesion of the IAN and the LCP. The incidence of implant placement outside the bony housing in the posterior mandibular region seems to be low because there are few case-reports published, but a higher perforation rate is suspected because this type of adverse event could have been unnoticed or unreported (5). In undercut types, the LCP during implant placement may lead to a severe hemorrhage, a persistent inflammation, or a possible infection which may result from dental implant that perforates the lingual oral mucosa and is then exposed to the oral environment (14). If left unattended, the infection might spread to the parapharyngeal and retropharyngeal space, leading to more severe complications, such as mediastinitis, mycotic aneurysm formation with possible subsequent rupture of the internal carotid artery, internal jugular vein thrombosis with septic pulmonary embolism or upper airway obstruction. Those complications may not occur immediately, however their insidious nature warrants more attention when planning surgeries in this area (5). Arterial hemorrhage in the posterior, lingual, mandibular region originated in the mylohyoid artery could be controlled by strong finger pressure at the point of bleeding or in the medial mandibular area just distal to the roots of the third molar. An attempt to ligate this artery would be difficult or impossible, and an artery dissection could complicate the situation even further (1). When arterial trauma occurs in this region of the oral cavity, hemorrhage can occur after a latency period, subsequently leading to airway obstruction and then death (14).

Panoramic radiography can be considered for primary evaluation in order to obtain information about the bone height and, to some extent, also information of horizontal distances (15). Panoramic radiographies give, however, only information in two dimensions, and have several disadvantages, such as image distortion and magnifications, which lead to inaccurate information (3,15).

Nickenig *et al.* (11) studied also the relationship between the position of the IAN and the prevalence and the depth of lingual undercuts. This author found that a deep position of the IAN was significantly associated with a lingual undercut, suggesting that a conventional panoramic radiography showing a deep position of the IAN could be an indication for a dental CT and also a guided implant insertion in order to prevent a lingual plate perforation. (11)

Dental CT has proven to be an excellent procedure for characterizing the anatomy and dental-related abnormalities of the jaw, the bone quality and morphology (2,7,16). It offers the additional possibility of multiplanar reconstructions in high quality and true-to-size hard copies. In dental implant therapy allows the appropriate choice of implant size and help to avoid injury of critical structures such de IAN (7). However, CT examinations are expensive and deliver a relatively high radiation dose to the patient. The most recent introduced imaging modality is the CBCT, which seems very promising with regard to pre-implant imaging (6,15,16).

Some authors like Kalpidis and Konstantinidis consider that routine preoperative CT or CBCT scanning is not necessary (9). Alternatively, at the time of implant treatment planning and surgery in the posterior mandible, careful preoperative digital palpation of the lingual mandibular surface, adequate reflection of the lingual mucoperiosteal flap, and full direct vision of the lingual cortex may have been assistance (5,9). When a significant lingual concavity is encountered, a CT or CBCT scan with a radiographic guide may be indicated preoperatively so that the implant angulation in relation to this anatomic limitation can be assessed (5,11). Furthermore, in order to avoid LCP during the preparation of the implant bed is very important to be aware of the resistance noticed by the blur when approaching the LC.

If wider implants (e.g., 5mm) are inserted in this area, the risk of perforation increases (6,14). In those cases that require the placement of all the implants parallel to each other within a jaw, the bone width that remains available is even smaller (6). When wider implants under specific conditions (e.g. parallel to each other) have to be inserted, Quirynen *et al.* (17) recommended the use of additional radiographic examinations, besides a panoramic view, for some bone morphologic categories (17). Furthermore, studies lend support to the use of implants of narrower diameter, in terms of clinical success, compared with 5.0mm diameter implants. Hence, in cases where significant lingual concavity exists, the use of narrower diameter implant such as 3.7 mm implant may be a viable alternative in this region of the oral cavity to avoid accidental perforation of the lingual plate (14). Chan *et al.* (5) recommended the use of tapered implants instead of parallel ones, or placing implants off- axially to avoid the concavity and restored with an angled abutment if there is a concern of lingual plate perforation after axial placement (5,11). In these cases bone augmentation should also be considered. (11) 

The U-type ridge mandibles present a potential increased risk of lingual cortical perforation during implant placement surgery. CBCT imaging provides an excellent delineation of the anatomy of the submandibular fossa and dental-related abnormalities of the jaw, the bone quality and morphology. This information is essential for the preoperative assessment of the posterior mandible in dental implants surgery.
